# Use of the tracheal bisecting line to differentiate upper pole from lower pole parathyroid adenomas: a PET/MR study using [^18^F]Fluorocholine

**DOI:** 10.1007/s12149-025-02105-9

**Published:** 2025-10-09

**Authors:** Junko Inoue Inukai, Bert-Ram Sah, Stephan Beintner-Skawran, Alexander Maurer, Simon A. Mueller, Grégoire B. Morand, Munenobu Nogami, Takamichi Murakami, Niels J. Rupp, Petra Petranović Ovčariček, Luca Giovanella, Martin W. Huellner

**Affiliations:** 1 Department of Nuclear Medicine, University Hospital Zurich and University of Zurich, Rämistrasse 100, 8091 Zurich, Switzerland; 2 Department of Radiology, Kobe University Graduate School of Medicine, 7-5-1, Kusunoki-Cho, Chuo-Ku, KobeHyogo, 650-0017 Japan; 3 Department of Diagnostic, Interventional, and Pediatric Radiology, Inselspital, University of Bern, Freiburgstrasse 20, 3010 Bern, Switzerland; 4 Department of Otorhinolaryngology, Head and Neck Surgery, University Hospital Zurich and University of Zurich, Rämistrasse 100, 8091 Zurich, Switzerland; 5 Division of Medical Imaging, Biomedical Imaging Research Center, University of Fukui, Matsuokashimoaizuki, Eiheiji, Yoshida, Fukui, 23-3910-1193 Japan; 6 Department of Pathology and Molecular Pathology, University Hospital Zurich and University of Zurich, Rämistrasse 100, 8091 Zurich, Switzerland; 7 Department of Oncology and Nuclear Medicine, University Hospital Center Sestre Milosrdnice, Vinogradska Cesta 29, 10000 Zagreb, Croatia; 8 School of Medicine, University of Zagreb, Ul. Radoslava Cimermana 88, 10000 Zagreb, Croatia; 9 Department of Nuclear Medicine, Gruppo Ospedaliero Moncucco SA, Clinica Moncucco, Via Soldino 5, 6900 Lugano, Switzerland

**Keywords:** Hyperparathyroidism, Fluorocholine, Positron emission tomography/computed tomography, Positron emission tomography/magnetic resonance imaging, Parathyroid glands, Trachea

## Abstract

**Purpose:**

Accurate preoperative localization of parathyroid adenomas is crucial in managing primary hyperparathyroidism (pHPT). Although [^18^F]Fluorocholine PET/CT and PET/MR have shown superior sensitivity over conventional imaging, distinguishing upper from lower pole adenomas remains challenging due to the invisibility of the recurrent laryngeal nerve. This study introduces the “tracheal bisecting line” (TBL), a novel anatomical landmark on axial PET/MR images, to aid in polarity differentiation.

**Methods:**

This retrospective study included 110 patients (128 adenomas) with biochemically confirmed pHPT who underwent [^18^F]Fluorocholine PET/MR at the University Hospital Zurich between December 2020 and October 2023. Adenomas were classified as upper (posterior to the TBL), lower (anterior), with mid-TBL cases (neither anterior nor posterior) treated as upper per default, according to evidence from surgical experience. Two board-certified readers independently assessed the images. Surgical findings served as the reference. Inter-reader agreement was evaluated using weighted kappa statistics, and diagnostic accuracy was calculated for all adenomas and subgroups: orthotopic, non-orthotopic, and intrathyroidal.

**Results:**

Among the 128 adenomas, 109 were orthotopic and 19 non-orthotopic (6 intrathyroidal). The inter-reader agreement was nearly perfect (*κ* = 0.99). The overall accuracy using the TBL was 0.945. The accuracy improved to 0.982 for orthotopic adenomas but decreased to 0.789 for non-orthotopic and 0.667 for intrathyroidal cases. Excluding intrathyroidal adenomas raised accuracy to 1.0 in non-orthotopic cases and 0.959 overall for non-intrathyroidal adenomas.

**Conclusions:**

The TBL is a reliable and reproducible landmark on [^18^F]Fluorocholine PET/MR for predicting parathyroid adenoma polarity, particularly in orthotopic and non-intrathyroidal cases. It may enhance surgical planning by improving anatomical clarity.

## Introduction

Primary hyperparathyroidism (pHPT) is an endocrine disorder characterized by excessive secretion of parathyroid hormone (PTH), often leading to hypercalcemia [1]. In most cases, the disorder is attributed to benign parathyroid adenomas, which cause the parathyroid glands to become enlarged and overactive. Surgical excision is the treatment of choice, and precise preoperative localization of the adenoma is critical for effective and minimally invasive surgery [2].

Conventional nuclear medicine imaging, such as [^99m^Tc]Tc-MIBI scintigraphy, has long been employed to localize parathyroid adenomas. Several studies have reported that the use of [^99m^Tc]Tc-MIBI single photon compute tomography (SPECT)/computed tomography (CT), 4D-CT, or 4D-magnetic resonance (MR) can improve the sensitivity for detecting parathyroid adenomas [3–8]. However, recent evidence suggests that [^18^F]Fluorocholine positron emission tomography (PET)/CT provides a higher degree of accuracy in detecting these lesions [9–13]. Moreover, the advent of PET/MR, which simultaneously acquires metabolic and high-resolution anatomical images, offers the potential for further improvements in localization accuracy [12–16] and was already proven useful in a variety of head and neck applications[17–20].

Despite these advancements, the precise determination of the adenoma's anatomical location—specifically, whether it represents an upper pole (superior gland) adenoma or a lower pole (inferior gland) adenoma (i.e., its polarity)—remains challenging. The superior glands originate from the fourth pharyngeal pouch and descend to their typical location on the posterior surface of the middle portion of the thyroid gland. In contrast, the inferior glands arise from the third pharyngeal pouch along with the thymus, migrating caudally along the lateral aspect of the thyroid to reach its lower pole. As demonstrated by these embryological migration pathways, the surgical distinction between upper and lower pole adenomas is determined by their spatial relationship to the recurrent laryngeal nerve. Specifically, upper pole adenomas are situated deep to this nerve. During surgical removal of upper pole adenomas, their proximity to the recurrent laryngeal nerve represents the primary concern, as this relationship poses a significant risk of nerve injury. Since this nerve cannot be visualized on cross-sectional imaging, we investigated whether the trachea could serve as a proxy for the nerve. The recurrent laryngeal nerve, a branch of the vagus nerve, loops beneath the subclavian artery on the right and under the aortic arch on the left. It then ascends bilaterally along the tracheoesophageal groove, passing near the cricothyroid joint before entering the larynx from the posterior aspect [21]. Given this anatomical course, the recurrent laryngeal nerve is generally expected to be located posterior to the bisecting line that divides the trachea into anterior and posterior halves. In this study, we propose the use of a “tracheal bisecting line,” drawn to separate anterior and posterior halves of the trachea, as a novel landmark for assessing the position of parathyroid adenomas. By applying this method during [^18^F]Fluorocholine PET/MR, we aimed to determine the reliability of differentiating between adenomas arising in the superior and inferior glands.

## Materials and methods

### *Patient*s

This retrospective study received ethical approval from our Institutional Review Board. Only patients with documented willingness to contribute their data to retrospective research were included.

The inclusion criteria for this study were as follows: patients who were clinically diagnosed with pHPT, underwent [^18^F]Fluorocholine PET/MR at the University Hospital Zurich between December 2020 and October 2023, and provided consent for the retrospective study (*n* = 125).

The exclusion criteria were as follows: patients who did not undergo parathyroid surgery after PET (*n* = 9), those in whom no adenoma was identified during surgery (*n* = 4), a patient where the adenoma was not visible on PET but was detected during surgery (*n* = 1), and a patient with a final diagnosis of tertiary hyperparathyroidism (tHPT) (*n* = 1). This condition typically arises in patients with longstanding secondary hyperparathyroidism, such as those with end-stage renal disease, and is often managed conservatively rather than surgically. Moreover, its underlying pathophysiology differs from that of primary hyperparathyroidism. Accordingly, this single case was excluded to preserve a homogeneous study population limited to surgically treated pHPT.

Since parathyroid adenoma and hyperplasia cannot be reliably differentiated on histopathology, the term “adenoma” is used throughout our manuscript to describe benign hyperfunctioning parathyroid glands causing hyperparathyroidism, as per convention at our institution.

### [^18^F]Fluorocholine PET/MR

The hybrid PET/MR scanner used in this study was a SIGNA PET/MR by GE HealthCare, operating at a magnetic field strength of 3.0 T.

Patients were injected with a standardized dose of 146.9 ± 14.3 MBq of [^18^F]Fluorocholine. Image acquisition started after 50 min. The PET images were corrected for random, dead time, scatter, and attenuation in a standardized way. PET image reconstruction was performed using 3D time-of-flight ordered subset expectation maximization with two iterations and 28 subsets (matrix size, 256 × 256 pixels; acquisition time, 3 min per bed position; 2 bed positions per patient; axial field of view, 153 mm) and block sequential regularized expectation maximization (BSREM, *β* value of 200). All readouts were done using the BSREM data sets.

Attenuation correction used an axially acquired T1-weighted Dixon-type sequence. The MR protocol covering the area from the tip of the head to the upper abdomen consisted of the following MR pulse sequences: a T1-weighted liver acquisition with volume acceleration-flexible (LAVA-flex) sequence, and a coronal T2-weighted sequence.

The regionalized diagnostic MR protocol of the head and neck and upper mediastinum consisted of the following MR pulse sequences: axial T1-weighted fast spin echo, coronal T2-weighted with short tau inversion recovery, axial T2-weighted with iterative decomposition of water and fat with echo asymmetry and least squares estimation, and axial T1-weighted LAVA-flex with reconstruction of in-phase and opposed-phase images.

No gadolinium contrast was used. Detailed PET/MR acquisition parameters have been described previously [14, 22].

### Image analysis

This study utilized the tracheal bisecting line (TBL), which divides the trachea into anterior and posterior halves on axial PET/MR images. Parathyroid adenomas demonstrating [^18^F]Fluorocholine uptake posterior to this line were classified as upper pole adenomas, while those located anteriorly were classified as lower pole adenomas, irrespective of their actual *Z*-axis level in relation to the thyroid gland. If an adenoma appeared to have an equal volume distributed between the anterior and posterior halves, it was categorized as mid-TBL. Per default, mid-TBL adenomas were classified as posterior, thus interpreted as upper pole adenomas. This classification was adopted to facilitate surgical planning, as such adenomas are presumed to have a comparably close anatomical relationship to the recurrent laryngeal nerve.

Two independent readers, both board-certified in radiology and nuclear medicine, reviewed the PET/MR images and categorized each adenoma as posterior, anterior, or mid-TBL based on its location relative to the TBL. The categorization was done using axial T2w-weighted PET/MR images.

### Statistical analysis

Inter-reader agreement on adenoma location was assessed using the kappa coefficient. This analysis was performed using MedCalc® Statistical Software version 20.218 (MedCalc Software Ltd., Ostend, Belgium; https://www.medcalc.org; (accessed on 26 March 2025)).

The surgical report was used as the reference standard to evaluate the diagnostic accuracy of PET/MR. Specifically, if an adenoma was documented as originating from the upper pole in the surgical report and it was classified as posterior or mid-TBL on PET/MR imaging, the interpretation was considered accurate. Likewise, if it was documented as originating from the lower pole and it was classified as anterior on imaging, the interpretation was considered accurate.

In addition, subgroup analyses were performed for different adenoma locations, including orthotopic adenomas only, non-orthotopic adenomas only, intrathyroidal parathyroid adenomas (ITPAs) within the non-orthotopic subgroup, and non-orthotopic adenomas excluding ITPAs.

Fisher’s exact test was used to assess whether there were statistically significant differences in diagnostic accuracy between subgroups, specifically orthotopic vs. non-orthotopic and orthotopic vs. intrathyroidal adenomas.

## Results

**Fig. 1:**
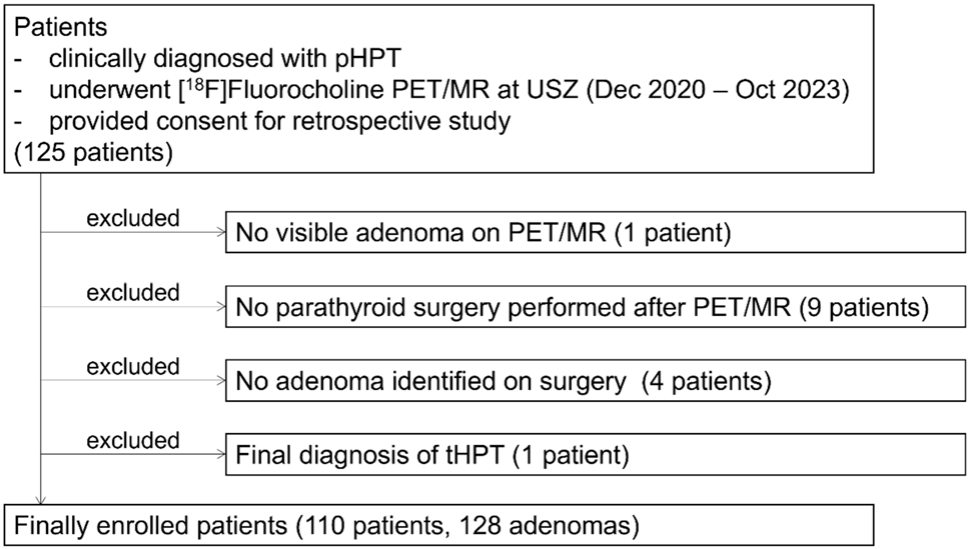
Flow chart of inclusion, exclusion, and final enrolment

**Fig. 2:**
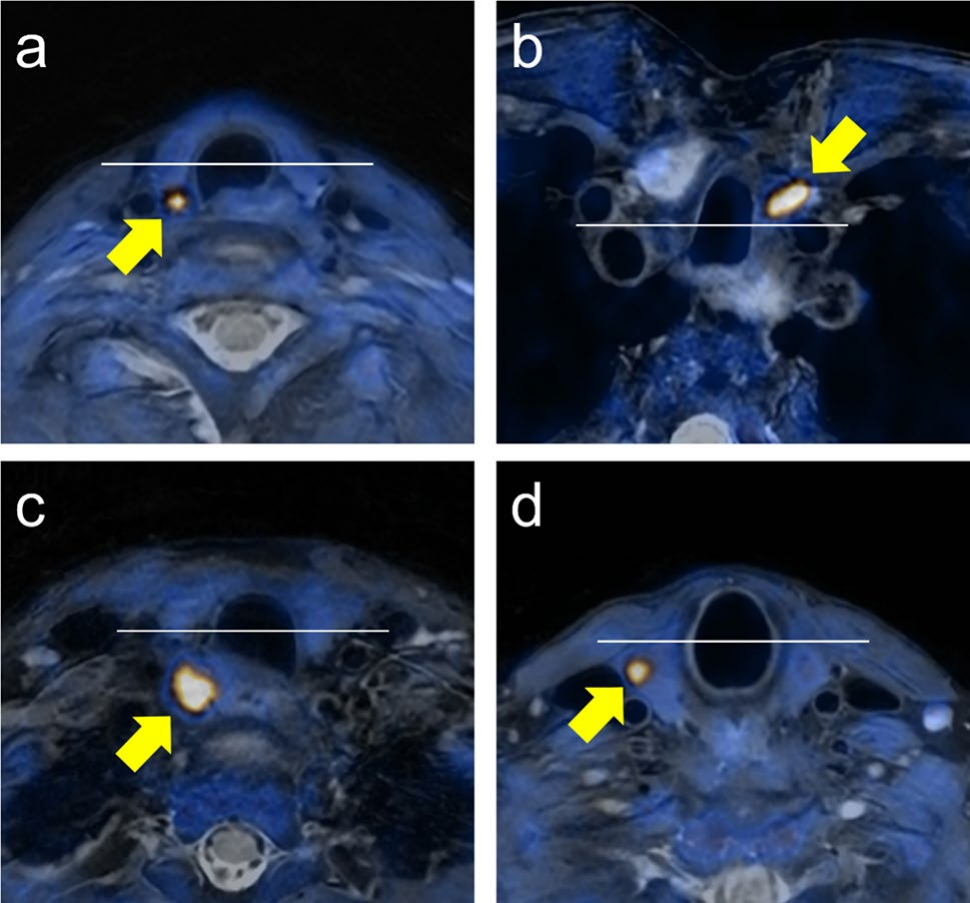
Representative T2-weighted fat-suppressed [^18^F]Fluorocholine PET/MR images of parathyroid adenoma location in relation to the TBL in four different subjects. **a** Orthotopic upper pole parathyroid adenoma located posterior to the TBL. **b** Orthotopic lower pole parathyroid adenoma located anterior to the TBL. **c** Overly descended upper pole parathyroid adenoma located posterior to the TBL. **d** Subcapsular intrathyroidal upper pole parathyroid adenoma located posterior to the TBL. White line: tracheal bisecting line (TBL); yellow arrows indicate parathyroid adenomas

A total of 128 parathyroid adenomas was identified in the 110 patients (on average 1.16 per patient, range 1 to 3) (Fig. 1**, **Table 1), with 109 orthotopic adenomas (85.2%) and 19 non-orthotopic adenomas (14.8%), the latter consisting of 7 overly descended adenomas (5.5%), 2 undescended adenomas (1.6%), 6 ITPA (thereof 2 subcapsular adenomas; 1.6%, and 4 intraparenchymal adenomas; 3.1%), and 4 major ectopias (3.1%) (Fig. 2).

**Table 1:** Demographic data of included subjects

Characteristic	All patients (*n* = 110)
Female sex, no. (%)	91 (82.7)
Age (years), mean ± SD	64.5 ± 13.2
Serum PTH level (pg/mL) before PET/MR, median (range)	109.5 (53.2–1391.0)

Almost perfect agreement was observed in the inter-reader assessment (weighted kappa = 0.99). In one discrepant case, one reader classified the adenoma as mid-TBL, while the other classified it as posterior. However, since both mid-TBL and posterior adenomas were interpreted as upper pole adenomas, this discrepancy did not affect the subsequent analysis of diagnostic accuracy. As a result, both reader 1 and reader 2 yielded the same final classification in the accuracy assessment.

The overall diagnostic accuracy for adenoma localization was 0.945 for both reader 1 and reader 2. When considering only orthotopic adenomas, the accuracy increased to 0.982. High accuracy (0.959) was maintained if ectopic adenomas arising inside the thyroid were excluded. In contrast, the accuracy of the proposed method was generally lower for non-orthotopic adenomas (0.789). Among these, the accuracy for intrathyroidal adenomas was further reduced to 0.667. However, when intrathyroidal adenomas were excluded, the accuracy for the remaining non-orthotopic adenomas increased to 1 (Table 2).

**Table 2:** Contingency table for different types of parathyroid adenomas as per location, showing adenoma designation as posterior or anterior to the TBL by the two readers, and parathyroid adenoma identity as upper or lower pole adenoma as per surgical report. Since parathyroid adenomas classified as “mid-TBL” were grouped with upper pole lesions during interpretation, the single case of reader disagreement did not alter the final anterior–posterior classification. As a result, both readers ultimately reached identical outcomes

Parathyroid adenoma type	*n*	Accuracy	Reference standard		Reader 1 and 2		Sum
					Posterior	Anterior	
All adenomas	128	0.945	Surgical report	Upper	84	2	86
				Lower	5	37	42
				Sum	89	39	128
Orthotopic adenomas	109	0.982	Surgical report	Upper	74	1	75
				Lower	1	33	34
				Sum	75	34	109
Non-orthotopic adenomas	19	0.789	Surgical report	Upper	11	1	12
				Lower	3	4	7
				Sum	14	5	19
Intrathyroidal adenomas	6	0.667	Surgical report	Upper	2	1	3
				Lower	1	2	3
				Sum	3	3	6
Non-orthotopic, extrathyroidal adenomas	13	1.000	Surgical report	Upper	8	0	8
				Lower	0	5	5
				Sum	8	5	13
All except intrathyroidal adenomas	122	0.959	Surgical report	Upper	82	1	83
				Lower	4	35	39
				Sum	86	36	122

Statistical comparison of diagnostic accuracy between subgroups revealed significant differences: orthotopic versus non-orthotopic (*p* = 0.004) and orthotopic versus intrathyroidal (*p* = 0.013).

## Discussion

This study hypothesized that the tracheal bisecting line will serve as a robust reference, thereby enhancing the accuracy of image interpretation and the surgeon's ability to plan targeted and effective surgical interventions. It not only seeks to refine the preoperative diagnostic process but also aims to contribute to improved patient outcomes through more precise surgical localization.

Further research and validation in larger cohorts will be essential to fully establish the clinical utility of this approach. Nonetheless, our initial findings provide promising evidence that the integration of PET/MR with innovative anatomical landmarks can significantly enhance the exact localization of parathyroid adenomas in primary hyperparathyroidism. Future studies may also assess the impact of TBL-guided localization on surgical outcomes, including operative time, complication rates, and cure rates.

The results of this study are particularly valuable for PET readers as they facilitate the creation of accurate reports. Although the polarity of adenomas is a key consideration in our analysis, it should be noted that surgeons are more concerned with lateralization (left/right sidedness) when planning surgical interventions, because the surgical access is one-sided. In addition, unilateral upper and lower parathyroids are reached through the same access. Since the recurrent laryngeal nerve serves as an important landmark for surgeons, the additional information obtained from PET with the TBL serving as a proxy for the nerve, may provide guidance on where to explore once the nerve is identified. The TBL is ill-suited as a direct surgical landmark, as the trachea remains covered by the thyroid gland during parathyroid surgery. Nonetheless, the preoperative distinction between upper and lower parathyroid adenomas remains valuable for surgical planning. In line with this surgical perspective, adenomas categorized as mid-TBL on imaging were by default interpreted as upper pole adenomas. This approach was chosen, because mid-TBL lesions are located comparably close to the recurrent laryngeal nerve, and classifying them as upper pole adenomas reflects the surgical priority of minimizing the risk of nerve injury during exploration. Particularly in challenging cases or when dealing with small adenomas, this distinction can guide the surgeon’s exploration after identifying the recurrent laryngeal nerve, helping to avoid inadvertent resection of healthy parathyroid tissue. The reliability of the TBL in re-operative settings, where anatomical distortion from previous surgeries and scarring is common, remains to be evaluated. Since the trachea is generally preserved and remains identifiable as a structure even in these cases, the TBL may still prove useful. Use of the TBL may be beneficial also in cases with unusual anatomy, where the usual anatomical landmarks may be altered [23]. The TBL offers valuable information for surgeons while being simpler and more broadly applicable than previously reported nomenclatures to classify parathyroid adenomas [24].

In addition, the use of the TBL may well aid in the correct identification of supernumerary glands, although this study does not provide any direct evidence for this assumption. However, the embryological basis for parathyroid positioning suggests that the TBL might help distinguish between superior and inferior supernumerary glands.

Importantly, this approach is likely applicable not only to PET/MR but also to PET/CT, as the trachea is reliably visualized on both modalities. While the internal carotid artery (ICA) might serve as a better anatomical proxy in some cases, it is easily visualized on non-enhanced MRI but can be inconsistently seen on non-enhanced CT (which is used in the majority of clinical [^18^F]Fluorocholine PET/CT examinations worldwide), whereas the trachea is consistently identifiable as anatomic structure.

Our proposed strategy also demonstrated utility in correctly attributing ectopic adenomas, with the exception of intrathyroidal parathyroid adenomas (ITPA). The thyroid gland represents a well-known location for ectopic parathyroid adenomas. ITPAs can originate from both ectopic superior and inferior parathyroid glands. However, inferior glands are believed to account for the majority of ITPAs due to their longer migration pathway during embryonic descent, which increases the likelihood of entrapment during the fusion of the lateral and medial thyroid lobes [25–28].

There is still ongoing debate regarding whether ITPAs are more commonly located in the upper or lower portion of the thyroid. While some reports suggest a higher prevalence in the upper pole [29], the majority of studies indicate that they are more frequently found in the lower portion of the thyroid [26–28]. For intrathyroidal adenomas, the suggested approach of using the tracheal bisecting Line to differentiate between upper and lower pole adenomas does not appear to be reliable. While it worked for the 2 subcapsular ITPAs in our cohort, it did not work well for the 4 truly intraparenchymal ITPAs. According to our findings, ITPAs are best interpreted as lower pole adenomas if they have a true intraparenchymal location. However, the number of ITPAs in this study was relatively low, which limits definitive conclusions. Besides, the identification of an intrathyroidal location might be accomplished easier with PET/MR than with PET/CT.

One limitation of this study is the relatively small sample size, particularly the limited number of intrathyroidal adenomas.

A second limitation is the rather small sample size for ITPA.

A third limitation is the potential introduction of selection bias due to the exclusion of cases in which the adenoma was not visualized on PET or not confirmed during surgery. These exclusions were necessary to ensure a reliable reference standard for evaluating the diagnostic accuracy of our method. However, we acknowledge that such exclusions may limit the generalizability of our findings, particularly in cases, where imaging findings are inconclusive or surgical confirmation is challenging.

Finally, a more refined classification of the comparably uncommon mid-TBL lesions may be beneficial in future studies.

## Conclusion

The TBL serves as an accurate reference to determine the polarity of parathyroid adenomas, even non-orthotopic ones, with the exception of intrathyroidal adenomas.

This method offers a significantly simpler, more practical, and reproducible alternative to previous imaging-based localization techniques, potentially enhancing surgical planning by providing an easy-to-apply landmark for predicting the location of parathyroid adenomas in relation to the recurrent laryngeal nerve.

## Data Availability

The data that support the findings of this study are not openly available due to reasons of sensitivity and are available from the corresponding author upon reasonable request.

## Abbreviations

CT: Computed tomography
ICA: Internal carotid artery
ITPA: Intrathyroidal parathyroid adenoma
MBq: Mega Becquerel
MRI: Magnetic resonance imaging
PET: Positron emission tomography
pHPT: Primary hyperparathyroidism
PTH: Parathyroid hormone
SD: Standard deviation
SUV_max_: Maximum standardized uptake value
TBL: Tracheal bisecting line
tHPT: Tertiary hyperparathyroidism
